# Epidemiology of tick-borne encephalitis in China, 2007- 2018

**DOI:** 10.1371/journal.pone.0226712

**Published:** 2019-12-26

**Authors:** Xiaojing Chen, Fan Li, Qikai Yin, Wenjing Liu, Shihong Fu, Ying He, Wenwen Lei, Songtao Xu, Guodong Liang, Shiwen Wang, Guang Yang, Xiaopeng Qi, Huanyu Wang

**Affiliations:** 1 Department of Viral Encephalitis, NHC Key Laboratory of Biosafety, National Institute for Viral Disease Control and Prevention, Chinese Center for Disease Control and Prevention, Beijing, China; 2 Department of Epidemiology, School of Medicine, Jinan University, Guangzhou, China; 3 State Key Laboratory for Infectious Disease Prevention and Control, Chinese Center for Disease Control and Prevention, Beijing, China; 4 Department of Basic Medical, School of Medicine, Qingdao University, Qingdao, China; 5 Office for Disease Control and Prevention, National Institute for Viral Disease Control and Prevention, Chinese Center for Disease Control and Prevention, Beijing, China; 6 CDC-WIV Joint Research Center for Emerging Diseases and Biosafety, Wuhan, China; 7 Center for Global Public Health, Chinese Center for Disease Control and Prevention, Beijing, China; Faculty of Science, Ain Shams University (ASU), EGYPT

## Abstract

Tick-borne encephalitis (TBE) is endemic to Europe and some Asian countries and is prevalent in northeast China. We analyzed the epidemiology of TBE in China from 2007 to 2018. A total of 3,364 TBE cases were reported in mainland China from 2007 to 2018, for an annual incidence of 0.09 to 0.44/100,000. Among the TBE cases, 89.92% were reported in forest areas (41.94% in DaXingAnLing, 8.70% in XiaoXingAnLing, and 39.21% in ChangBaiShan) in northeast China. The TBE cases were primarily male with a proportion of 67.15% (2,259/3,364 cases) and in 40–49-year age group with a proportion of 31.89% (1,073/3,364 cases). The epidemiology of TBE differed slightly among the three forest regions. Domestic workers and forestry workers accounted for the most of the TBE cases in DaXingAnLing, and domestic workers and farmers in XiaoXingAnLing and ChangBaiShan, respectively. The TBE cases mainly occurred from April to August with a peak in June. The TBE laboratory confirmed rate in DaXingAnLing (84.14%, 1,189/1,413 cases) was highest, compared with XiaoXingAnLing and ChangBaiShan (13.99% and 11.37%, respectively). Moreover, the hospital with the highest laboratory confirmed rate (88.01%, 1,336/1,518 cases) was Inner Mongolia Forestry General Hospital of DaXingAnling region. Systematic enhanced TBE surveillance and a vaccination program are needed to improve the laboratory confirmed rate and reduce the incidence of TBE in northeast China.

## Introduction

Tick-borne encephalitis (TBE) is transmitted by ticks carrying the tick-borne encephalitis virus (TBEV), which invades the central nervous system and causes serious morbidity. There is no antiviral therapy for TBE and so induction of active immunity is the main preventive measure[1, 2].TBEV is distributed widely in Europe and Asia and is endemic to 27 European countries and at least four Asian countries; 10,000–12,000 cases of TBE occur annually worldwide[3, 4].

TBEV is a member of the genus *Flavivirus*, family *Flaviviridae* and has a genome of approximately 11 kb[5]. TBEV is classified into the European (TBEV-Eu), Siberian (TBEV-Sib), and Far Eastern (TBEV-FE) subtypes^[^^1^^]^. Recently, the new subtypes Baikalian (TBEV-Bkl), which diverged from TBEV-Sib, has been proposed[6] and Himalayan (Him-TBEV) subtype has been identified in wild rodents[7].TBEV-Eu is predominantly found in Europe; TBEV-Sib in Siberia, the Baltic, and northern Finland; and TBEV-FE in east Asia[8].

The distribution of TBE in China is closely related to the distribution of its tick vectors[9]. Since its discovery in 1942, TBE cases have occurred mainly in the endemic regions in northeast China[10–12]. TBEV-FE is endemic in northern China and is transmitted by *Ixodes persulcatus*[11, 13, 14]. Forest areas in DaXingAnLing, XiaoXingAnLing, and ChangBaiShan are the typical TBE epidemic areas of northeast China. Moreover, TBE is intermittently reported in Xinjiang, where TBEV has been detected. Yunnan and Tibet provinces may also be a natural focus based on serology data[12, 15, 16].

Since it is not a notifiable infectious disease in China, surveillance of TBE has been lacking. Therefore, we carried out an epidemiological analysis of TBE in China using cases reported from China Information System for Diseases Control and Prevention over the past decade.

## Materials and methods

### 1. Ethics statement

The ethical approval of this study was determined by the Chinese Center for Disease Control and Prevention, that the collection of data from TBE cases was part of continuing public health surveillance of an infectious disease and was exempt from institutional review board assessment.

### 2. Case definition

According to the national guidelines, the diagnostic criteria for a clinically- diagnosed TBE case is based on the presence of clinical symptoms (such as acute fever, headache, vomiting, and/or typical central nervous system symptoms) in connection with exposure to forest during spring or summer, or a history of tick bite. A laboratory-confirmed TBE case is defined as the presence of increased anti-TBEV IgM and IgG levels, an at least fourfold increase in the level of anti-TBE antibody between acute and convalescent serum samples, or a positive result for TBEV RNA by PCR[8, 17].

### 3. Data collection and management

Although TBE is not a notifiable disease, clinical TBE cases diagnosed at medical institutions have been reported to the Chinese Information System for Diseases Control and Prevention (CISDCP) by the majority of provinces since 2002. The reported numbers of TBE cases and demographic information (such as age and gender) were obtained from CISDCP from January 2007 to December 2018. We divided the study area into DaXingAnLing, XiaoXingAnLing, ChangBaiShan, and other regions based on the geographic characteristics. Using the national land use data (grid) of 2015, we extracted areas of forest land, shrub forest, sparse forest land, and other forest land, and superimposed it on the national DEM data (raster data). We extracted areas of altitude ≥ 500 m and areas of forest land with mountainous terrain and overlaid them on a map of the county-level jurisdictional boundary in the northeast region. The counties with high forest coverage were extracted as the rough boundary of these three regions.

### 4. Epidemiological characteristics

**Fig 1:**
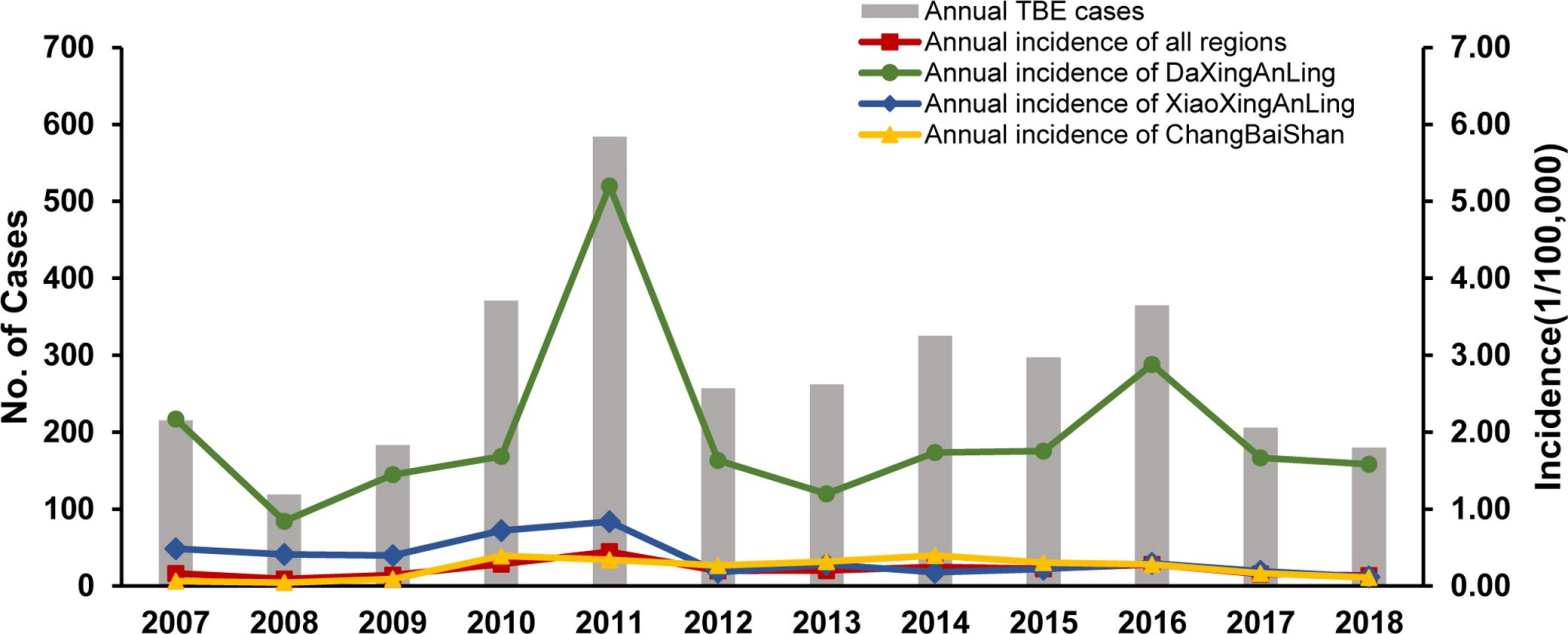
Annual number of cases and incidence of TBE from 2007 to 2018. Bars, annual TBE cases; red curve, annual incidence of all regions; green curve, annual incidence of DaXingAnLing; blue curve, annual incidence of XiaoXingAnLing; yellow curve, annual incidence of ChangBaiShan.

**Fig 2:**
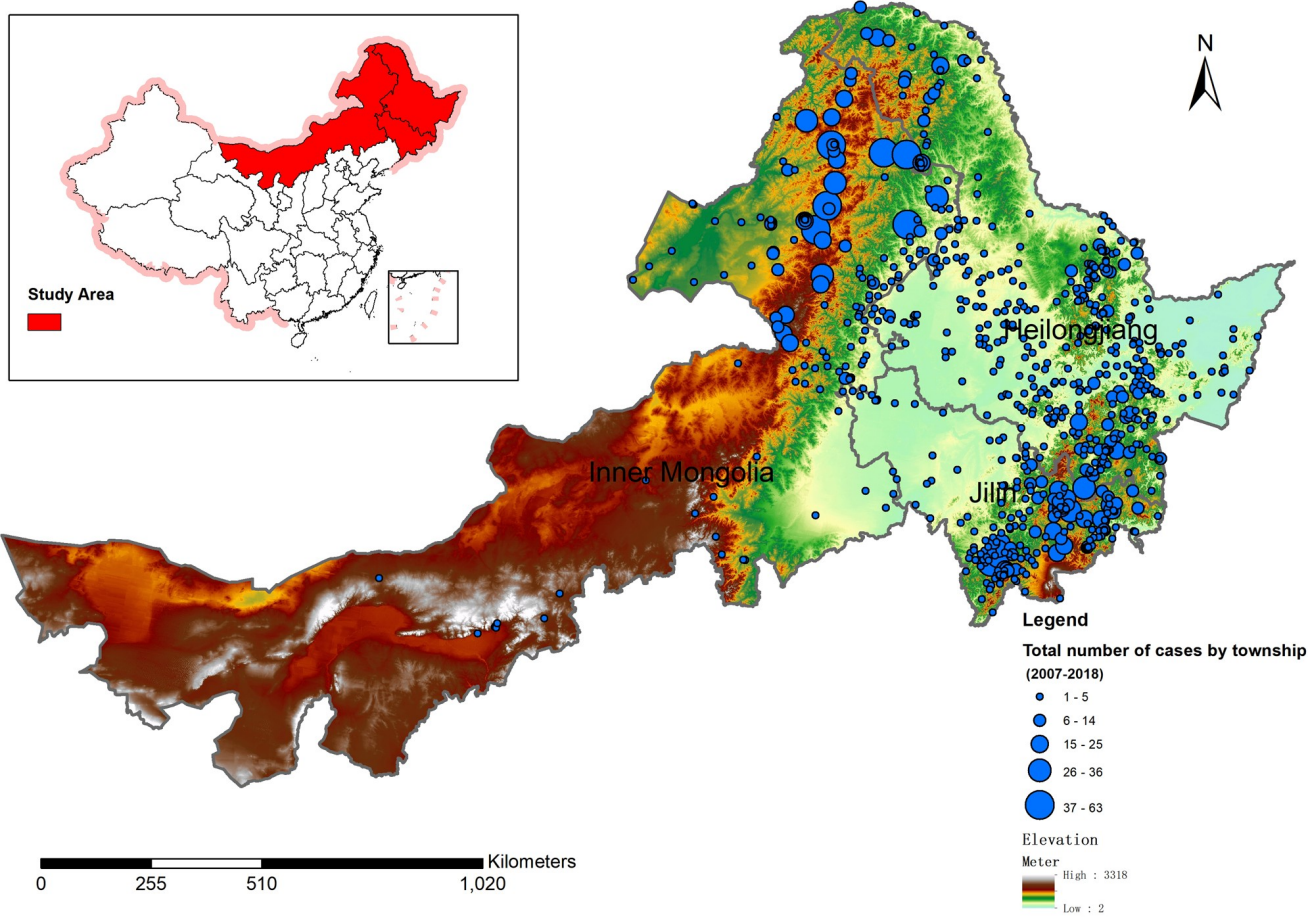
TBE case distribution on a topographic map. Red, endemic areas of TBE, concentrated in northeast China; blue circles, number of cases; background color, altitude. TBE cases were mainly distributed in northeast China at altitude > 500 m, including in DaXingAnLing, XiaoXingAnLing, and ChangBaiShan.

**Fig 3:**
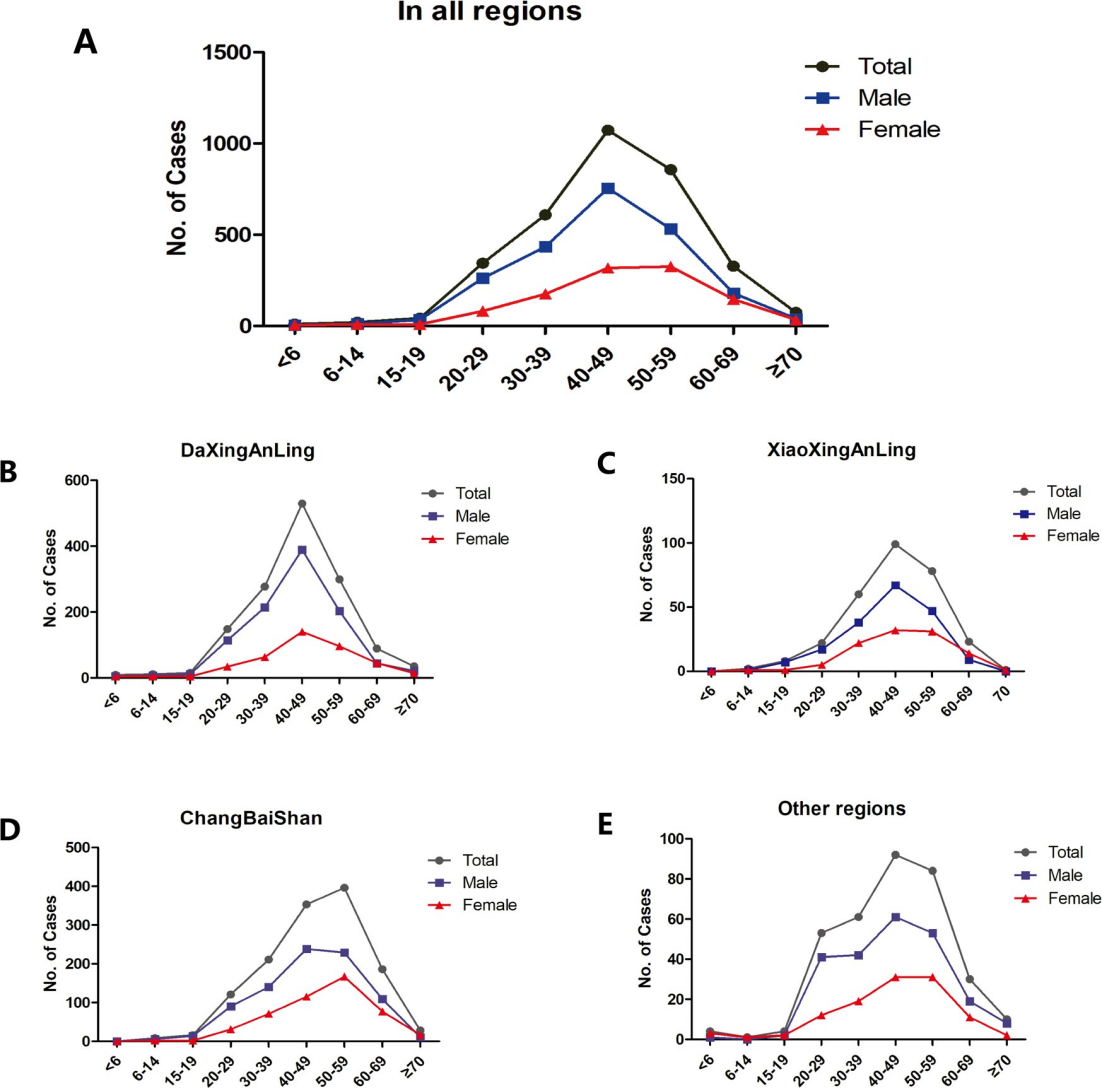
Gender and age distribution of TBE cases from 2007 to 2018. TBE cases by age group and gender. Black curve, total cases; blue curve, male cases; red curve, female cases. (A) TBE cases by age group and gender in all regions(B) in DaXingAnLing, (C) in XiaoXingAnLing, (D) in ChangBaiShan. (E) and in other regions.

**Fig 4:**
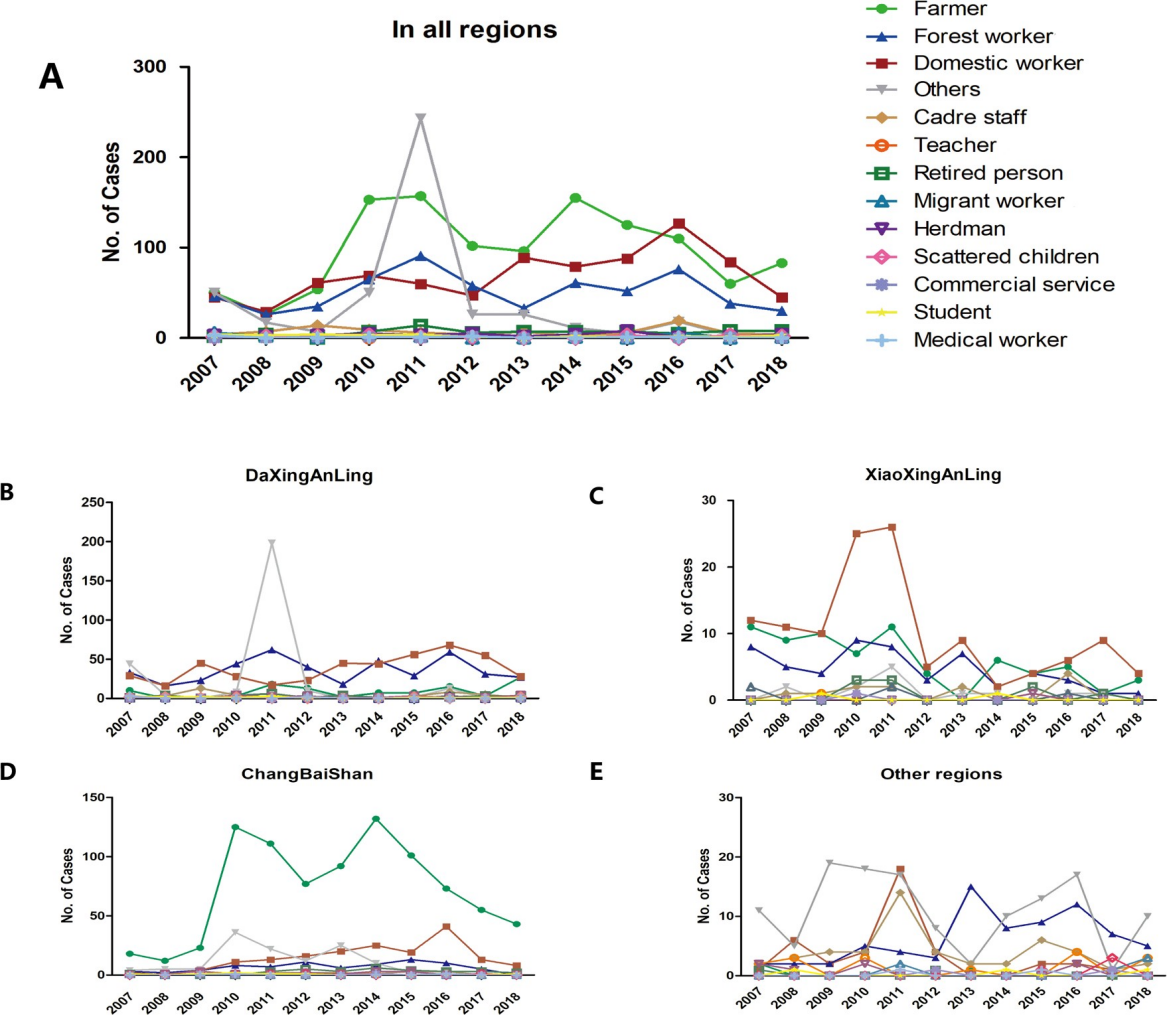
Occupations of TBE cases from 2007 and 2018. Annual variation in occupations (A) in all regions, (B) in DaXingAnLing, (C) in XiaoXingAnLing, (D) in ChangBaiShan, (E) and in other regions.

**Fig 5:**
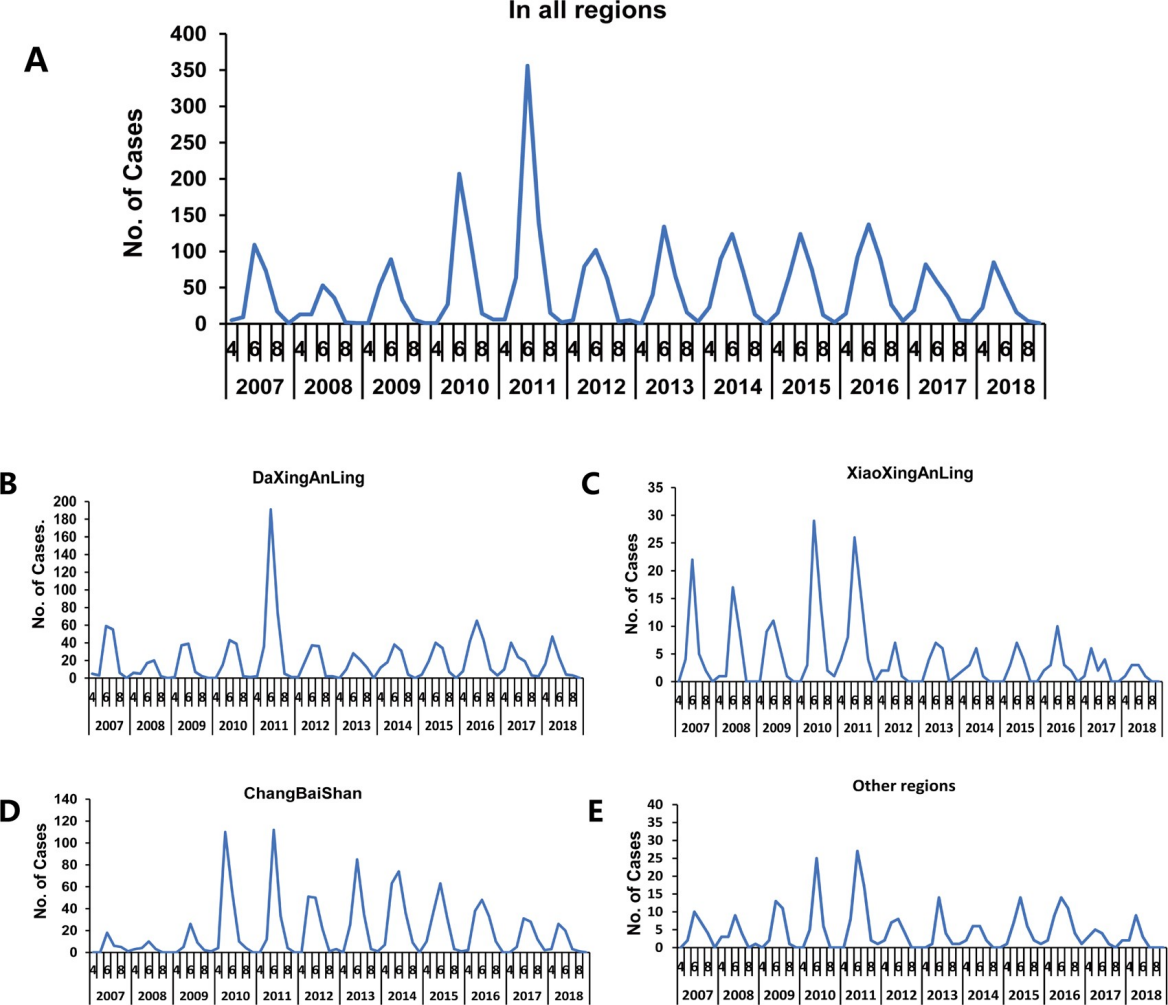
Monthly distribution of TBE cases from 2007 to 2018. (A) in all regions, (B) in DaXingAnLing, (C) in XiaoXingAnLing, (D) in ChangBaiShan, (E) and in other regions.

**Fig 6:**
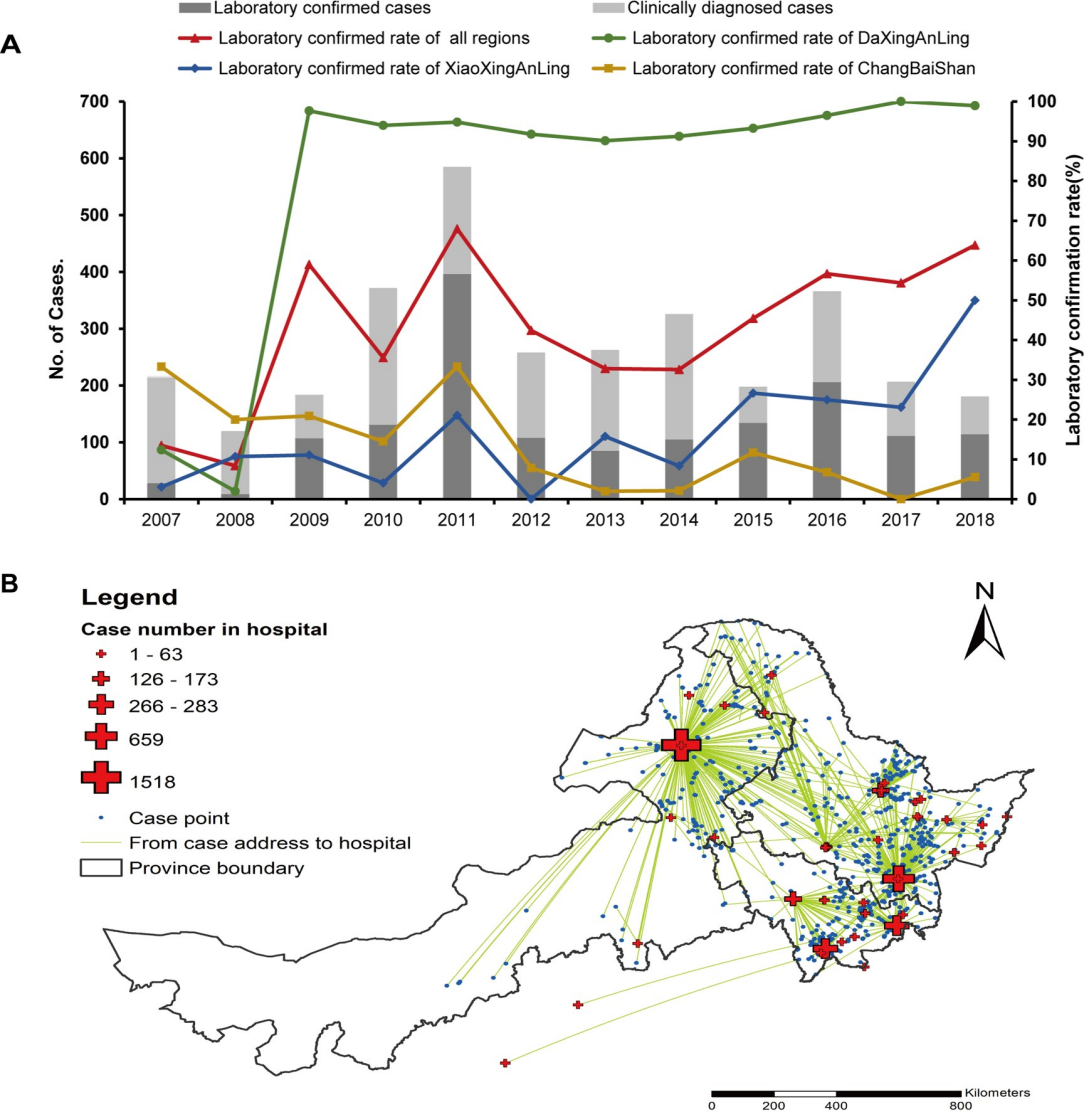
Case reports of TBE from 2007 to 2018. (A) Laboratory confirmed rate. Bars, light grey and dark grey, clinically confirmed cases and laboratory confirmed cases; red curve, annual laboratory confirmed rate of all regions; green curve, annual laboratory confirmed rate of DaXingAnLing; blue curve, annual laboratory confirmed rate of XiaoXingAnLing; yellow curve, annual laboratory confirmed rate of ChangBaiShan.(B) Distribution of hospitalized cases. Blue dot, case; cross, hospital; green line, route from the residence of a case to the hospital.

The annual incidence and mortality, the cumulative number of cases from 2007 to 2018, and the epidemic trend and seasonal characteristics of TBE were analyzed using Microsoft Excel™ 2008, SPSS (v. 24.0, IBM Crop, Armonk, NY), and ArcGIS (v. 10.0, ESRI, Redlands, CA) software (Figs 1, 2, 3, 4, 5 and 6 and S1 Table). A value of *P* < 0.05 by chi squared test was taken to indicate statistical significance.

## Results

### 1. Incidence of TBE

From 2007 to 2018, 3364 cases were reported with 19 deaths in China, for an annual incidence in all regions of 0.09 to 0.44/100,000. The annual incidence of DaXingAnLing, XiaoXingAnLing and ChangBaiShan was from 0.84 to 5.20/100,000, 0.12 to 0.84/100,000 and 0.04 to 0.39/100,000, respectively (Fig 1).

### 2. Geographic distribution of TBE

TBE cases were mainly distributed in the DaXingAnLing (1,413, 41.94%), XiaoXingAnLing (293, 8.70%), and ChangBaiShan (1,319, 39.21%) regions of northeast China. These regions have an altitude of > 500 m. Only 10.08% (339 cases) of the TBE cases occurred at altitude of < 500 m (S1 Table and Fig 2). Cases were primarily male, the male percentage of TBE cases in DaXingAnLing, XiaoXingAnLing, and ChangBaiShan were 71.34%, 63.48%, and 63.45%, respectively. TBE cases peaked in the 40–49-year age group. The median age of the TBE cases in ChangBaiShan, DaXingAnLing, and XiaoXingAnLing was 49, 44, and 46 years, respectively (S1 Table and Fig 3A). In DaXingAnLing, XiaoXingAnLing, and the other regions, TBE cases peaked in the 40–49 years of age in both male and female groups (Fig 3B, 3C and 3E). In ChangBaiShan, TBE cases peaked in females and males with 50–59 and 40–49-year age groups, respectively (Fig 3D).

### 3. Occupational distribution of TBE

Farmers (34.80%), domestic workers (24.46%), and forestry workers (18.16%) accounted for the top three of TBE cases from 2007 to 2018 (S1 Table and Fig 4A). In 2011, a large number of TBE cases in DaXingAnLing were of unknown occupation. The number of domestic workers in DaXingAnLing increased from 2011 to a peak in 2016 (Fig 4B). In XiaoXingAnLing occupational morbidity was stable and relatively low (with minor fluctuations from 2009 to 2011), except for domestic workers (Fig 4C). Farmers comprised the largest proportion of TBE cases in ChangBaiShan (S1 Table and Fig 4D). The distribution of the above three occupations varied among the regions (chi-squared test, *P* < 0.001). In DaXingAnLing and XiaoXingAnLing, the majority of TBE cases were found among domestic workers (32.13% and 41.98%, respectively), but the majority were farmers in ChangBaiShan (65.35%; S1 Table).

### 4. Monthly distribution of TBE

From 2007 to 2018, TBE cases were concentrated from April to August; the peak month in most years was June, but it was May in 2017 and 2018, in all regions and in the individual regions (Fig 5A). The number of TBE cases increased in June of 2011 in DaXingAnLing, and significantly increased in May and June of 2010 and 2011 in ChangBaiShan (Fig 5B and 5D). In XiaoXingAnLing, the number of TBE cases fluctuated obviously in 2007, 2008, 2010, and 2011 (Fig 5C).

### 5. Case reports

The national laboratory confirmed rate of TBE was 13.48% and 8.40% in 2007 and 2008, and sharply increased from 2009 with 59.01% to a peak in 2011 with 67.98%, mainly due to the increase in DaXingAnLing (84.14%, 1,189/1,413) (Fig 6A). The laboratory confirmed rate in DaXingAnLing was 84.14% (1,189/1,413). In DaXingAnLing, cases were mainly confirmed by Inner Mongolia Forestry General Hospital. During 2007 to 2018, a total of 1518 cases (45.42% of the national total) were reported by this hospital, with the laboratory confirmed rate of 88.01% (1,336/1,518). In XiaoXingAnLing, the laboratory confirmed rate was 13.99% (41/293). In ChangBaiShan, the laboratory confirmed rate was 11.37% (150/1,319), a total of 659 cases were reported in the Mudanjiang Forestry Center Hospital, at which laboratory confirmed rate was 19.12% (126/659) (S1 Table and Fig 6B).

## Discussion

The annual incidence of TBE in 2007 to 2018 was from 0.09 to 0.44/100,000. TBE cases were mainly distributed in northeast China, in DaXingAnLing and ChangBaiShan. TBE occurred from April to August, with a peak in May or June. TBE cases were predominantly found in men, 40–49 years of age, who worked as farmers, domestic workers, and forestry workers. The laboratory confirmed rate of TBE cases in all regions were less than 14% in 2007 and 2008 and sharply increased after 2009, mainly due to the increase in DaXingAnLing, 45.42% (1,518/3,364) of the TBE cases in all regions and 84.14% (1,189/1,413) of the DaXingAnLing TBE cases were reported in Inner Mongolia Forestry General Hospital.

TBE is an emerging health threat that is spreading in many parts of Europe and eastern Asia[18]. The main endemic regions of TBE in China are DaXingAnLing, XiaoXingAnLing and ChangBaiShan[15]. ChangBaiShan (altitude > 1,000 m) is cold and humid. The main vector of TBEV is *I*. *persulcatus*. The extensively forested XiaoXingAnLing (altitude 400–600 m) also has a cold and humid climate. These two areas have theropencedrymion vegetation comprising *Pinus koraiensis*, *Abies nephrolepis*, and *Picea jezoen*. In DaXingAnLing, a forest region of 100,000 km^2^ with a cold and humid climate, the forest vegetation comprises *Larix grnelinii* as well as the theropencedrymion. Compared to ChangBaiShan, *I*. *persulcatus* is less prevalent in DaXingAnLing; however, it is the dominant tick species (70% of the total)[12, 17]. Vegetation can provide a suitable living environment for ticks. The tick density is dependent on the type and structure of the forest; it is highest in mixed and deciduous forests[19]. In addition, some areas of China in which no cases have been reported are potential TBE regions[12], and so continual monitoring of TBEV is needed.

The high-risk groups for TBE are forestry workers and those engaged in outdoor agricultural work, who are predominantly male. However, domestic workers are also affected by TBE. In DaXingAnLing, the number of domestic workers increased from 2011 to 2016, possible due to increased participation in outdoor activities (*e*.*g*., hiking, biking, and mushroom picking)[20]. Outdoor activities involve contact with nature, especially with grass, bushes, and shrubbery, which increases the risk of tick bites and of contracting an infection [21]. Further study should focus on investigating the rate of vaccination in these areas, and the populations of affected areas should insist on vaccination.

The incidence of TBE peaked from April to August and the peak months were May and June, as reported previously[19]. In Europe, most TBE cases occur from June to September[15, 22]. The incidence of TBE usually peaks 2 weeks after the peak tick activity, which occurs in May. During June and July, when the average diurnal temperature reach 20°C, the density of ticks decreases and cases are rare after September[12, 15].

The laboratory confirmed rate of TBE cases in China sharply increased after 2009 and hospitalized cases were concentrated in Inner Mongolia Forestry General Hospital. Founded in 1956, Inner Mongolia Forestry General Hospital is the most northerly general hospital in China and is located in Hulunbuir grassland at the DaXingAnLing intersection of Yakeshi City, of which diagnosis, treatment and research of TBE and Lyme disease are at an advanced level in China.

As TBE is not a notifiable infectious disease in China, its incidence is likely to be under-reported. The disease system is based on discharge diagnosis without follow-up, death after discharge is not included, and the mortality seemed to be lower than expected. In addition, 33 emerging tick-borne agents have been identified in mainland China[23, 24]. Routine surveillance for tick borne diseases in China led to the identification of a patient from the town of Alongshan who had a febrile illness with an unknown cause, and found to be a new segmented and likely tick-borne virus, ALSV, has clinical manifestations similar to TBEV infection[25]. Thus, laboratory confirmation of clinically suspected cases is needed to better understand the incidence and disease burden of TBE in China. And also, enhanced vector-based and hospital-based sentinel TBE surveillance, and a vaccination program are needed to improve the laboratory confirmed rate and reduce the incidence and disease burden of TBE in China.

## Conclusion

We report here the epidemic characteristics of TBE according to the distribution of its natural region. The overall TBE notification rate remained stable from 2007 to 2018. In China, TBE is mainly distributed in DaXingAnLing and ChangBaiShan and was concentrated by Inner Mongolia Forestry General Hospital. Intensified hospital surveillance and promotion of TBE vaccination are needed to improve the laboratory confirmed rate and reduce the incidence and distribution of TBE in northeast China.

## Supporting information

S1 Table Epidemiologic features of TBE in three regions of China, 2007–2018. (DOCX)

## References

[1] Lindquist L, Vapalahti O. Tick-borne encephalitis. Lancet (London, England). 2008;371(9627):1861–71. Epub 2008/06/03. 10.1016/s0140-6736(08)60800-4 18514730

[2] Kaiser R. Tick-borne encephalitis. Der Nervenarzt. 2016;87(6):667–80. Epub 2016/05/27. 10.1007/s00115-016-0134-9 27225401

[3] Vaccines against tick-borne encephalitis: WHO position paper. Releve epidemiologique hebdomadaire. 2011;86(24):241–56. Epub 2011/06/15. 21661276

[4] Süss J. Epidemiology and ecology of TBE relevant to the production of effective vaccines. Vaccine. 2003;21:S19–S35. 10.1016/s0264-410x(02)00812-5 12628811

[5] Fuzik T, Formanova P, Ruzek D, Yoshii K, Niedrig M, Plevka P. Structure of tick-borne encephalitis virus and its neutralization by a monoclonal antibody. Nature communications. 2018;9(1):436 Epub 2018/02/01. 10.1038/s41467-018-02882-0 29382836 PMC5789857

[6] Kovalev SY, Mukhacheva TA. Reconsidering the classification of tick-borne encephalitis virus within the Siberian subtype gives new insights into its evolutionary history. Infection, genetics and evolution: journal of molecular epidemiology and evolutionary genetics in infectious diseases. 2017;55:159–65. Epub 2017/09/19. 10.1016/j.meegid.2017.09.014 28919548

[7] Dai X, Shang G, Lu S, Yang J, Xu J. A new subtype of eastern tick-borne encephalitis virus discovered in Qinghai-Tibet Plateau, China. Emerging microbes & infections. 2018;7(1):74 10.1038/s41426-018-0081-6 29691370 PMC5915441

[8] Bogovic P, Strle F. Tick-borne encephalitis: A review of epidemiology, clinical characteristics, and management. World journal of clinical cases. 2015;3(5):430–41. Epub 2015/05/20. 10.12998/wjcc.v3.i5.430 25984517 PMC4419106

[9] Gao X, Nasci R, Liang G. The neglected arboviral infections in mainland China. PLoS neglected tropical diseases. 2010;4(4):e624 Epub 2010/05/04. 10.1371/journal.pntd.0000624 20436960 PMC2860493

[10] Weiming B, Xiaoyan B, Houpei D. A regionalisation study of Natured epidemic areas of forest encephalitis. Journal of Capital Normal University. 1997;18(2):100–3. Epub 1997–06.

[11] Xueling H, Lili L. Research progess of clinical epidemiology on forest encephalitis(in Chinese). Medical Journal of National Defending Forces in Northwest China. 2018;29(3):148–51. Epub 2018-3-30.

[12] Lu Z, Broker M, Liang G. Tick-borne encephalitis in mainland China. Vector borne and zoonotic diseases (Larchmont, NY). 2008;8(5):713–20. Epub 2008/10/08. 10.1089/vbz.2008.0028 18837668

[13] He X, Zhao J, Fu S, Yao L, Gao X, Liu Y, et al Complete Genomic Characterization of Three Tick-Borne Encephalitis Viruses Detected Along the China-North Korea Border, 2011. Vector borne and zoonotic diseases (Larchmont, NY). 2018;18(10):554–9. 10.1089/vbz.2017.2173 29742014

[14] Shi J, Hu Z, Deng F, Shen S. Tick-Borne Viruses. Virologica Sinica. 2018;33(1):21–43. Epub 2018/03/15. 10.1007/s12250-018-0019-0 29536246 PMC5866268

[15] Xing Y, Schmitt HJ, Arguedas A, Yang J. Tick-borne encephalitis in China: A review of epidemiology and vaccines. Vaccine. 2017;35(9):1227–37. Epub 2017/02/06. 10.1016/j.vaccine.2017.01.015 28153343

[16] Zhang G-L, Liu R, Sun X, Zheng Y, Liu X-M, Zhao Y, et al Investigation on the endemic foci of new emerged tick-borne encephalitis in Charles Hilary, Xinjiang. Zhonghua liu xing bing xue za zhi = Zhonghua liuxingbingxue zazhi. 2013;34(5):438–42. Epub 2013/09/11. 24016430

[17] Sun RX, Lai SJ, Yang Y, Li XL, Liu K, Yao HW, et al Mapping the distribution of tick-borne encephalitis in mainland China. Ticks and tick-borne diseases. 2017;8(4):631–9. Epub 2017/05/04. 10.1016/j.ttbdis.2017.04.009 28461151

[18] Hotez PJ, Bottazzi ME, Strych U, Chang L-Y, Lim YAL, Goodenow MM, et al Neglected Tropical Diseases among the Association of Southeast Asian Nations (ASEAN): Overview and Update. PLoS neglected tropical diseases. 2015;9(4). 10.1371/journal.pntd.0003575 PubMed PMID: WOS:000354972200010. 25880767 PMC4400050

[19] Li Y, Wang J, Gao M, Fang L, Liu C, Lyu X, et al Geographical Environment Factors and Risk Assessment of Tick-Borne Encephalitis in Hulunbuir, Northeastern China. International journal of environmental research and public health. 2017;14(6). Epub 2017/06/08. 10.3390/ijerph14060569 28587151 PMC5486255

[20] Haditsch M, Kunze U. Tick-borne encephalitis: a disease neglected by travel medicine. Travel medicine and infectious disease. 2013;11(5):295–300. Epub 2013/08/07. 10.1016/j.tmaid.2013.07.003 23916617

[21] Kunze U. TBE—awareness and protection: the impact of epidemiology, changing lifestyle, and environmental factors. Wiener medizinische Wochenschrift (1946). 2010;160(9–10):252–5. Epub 2010/07/16. 10.1007/s10354-010-0798-x 20632154

[22] Beaute J, Spiteri G, Warns-Petit E, Zeller H. Tick-borne encephalitis in Europe, 2012 to 2016. Euro surveillance: bulletin Europeen sur les maladies transmissibles = European communicable disease bulletin. 2018;23(45). Epub 2018/11/15. 10.2807/1560-7917.es.2018.23.45.1800201 30424829 PMC6234529

[23] Fang LQ, Liu K, Li XL, Liang S, Yang Y, Yao HW, et al Emerging tick-borne infections in mainland China: an increasing public health threat. The Lancet Infectious diseases. 2015;15(12):1467–79. Epub 2015/10/11. 10.1016/S1473-3099(15)00177-2 26453241 PMC4870934

[24] Wu XB, Na RH, Wei SS, Zhu JS, Peng HJ. Distribution of tick-borne diseases in China. Parasites & vectors. 2013;6:119 Epub 2013/04/27. 10.1186/1756-3305-6-119 23617899 PMC3640964

[25] Wang ZD, Wang B, Wei F, Han SZ, Zhang L, Yang ZT, et al A New Segmented Virus Associated with Human Febrile Illness in China. The New England journal of medicine. 2019;380(22):2116–25. 10.1056/NEJMoa1805068 31141633

