# Supplementary material for: Epidemiology of tick-borne encephalitis in China, 2007- 2018
Source: PLoS One. 2019 Dec 26;14(12):e0226712. doi: 10.1371/journal.pone.0226712 (PMC6932775; doi:10.1371/journal.pone.0226712)
Supplement: S1 Table — (DOCX) [file pone.0226712.s001.docx]

|  | In all regions | DaXingAnLing | XiaoXingAnLing | ChangBaiShan | Other regions |
| --- | --- | --- | --- | --- | --- |
| Total cases（n,%） | 3364 | 1413(41.94) | 293(8.70) | 1319(39.21) | 339(10.08) |
| Laboratory-confirmed cases(n,%) | 1546(45.96) | 1189(84.14) | 41(13.99) | 150(11.37) | 166(49.26) |
| Male (n,%) | 2259(67.03) | 1009(71.40) | 186(63.48) | 837(63.45) | 227(66.96) |
| Occupation(n,%) |  |  |  |  |  |
| Farmers | 1171(34.80) | 107(7.57) | 71(24.23) | 862(65.35) | 131(38.64) |
| Domestic workers | 823(24.46) | 454(32.13) | 123(41.98) | 172(13.04) | 74(21.83) |
| Forest workers | 611(18.16) | 430(30.43) | 55(18.77) | 78(5.91) | 48(14.16) |
| Age |  |  |  |  |  |
| Media (range) | 45.05(0.1-97) | 44(0.1-97) | 46(9-76) | 49(6-81) | 45(1-87) |
| Mean(SD) | 46.00(12.76) | 43.70(12.29) | 44.51(11.59) | 46.96(12.86) | 43.68(14.31) |
| Epidemic months | 4-8 | 4-8 | 4-8 | 4-8 | 4-7 |

**S1 Table. Epidemiologic features of TBE in three regions of China, 2007–2018.**
